# Reassignment of the Structures of Products Produced by Reactions of the Product Believed To Be 2-(1-Phenyl-2-Thiocyanatoethylidene)-malononitrile with Electrophiles

**DOI:** 10.3390/molecules16053456

**Published:** 2011-04-26

**Authors:** Saleh Mohammed Al-Mousawi, Moustafa Sherief Moustafa, Mohamed Hilmy Elnagdi

**Affiliations:** Department of Chemistry, Faculty of Science, University of Kuwait, Safat, 13060, P.O. Box 12613, Kuwait

**Keywords:** 2-(1-phenyl-2-thiocyanatoethylidene)malononitrile, thiazole, diaminopyrazole, pyrazolo[1,5-α] pyrimidinediamine

## Abstract

The reactivity of the product believed to be 2-(1-phenyl-2-thiocyanato-ethylidene)malononitrile toward a variety of electrophilic and nucleophilic reagents is reported.

## 1. Introduction

**Scheme 1:**
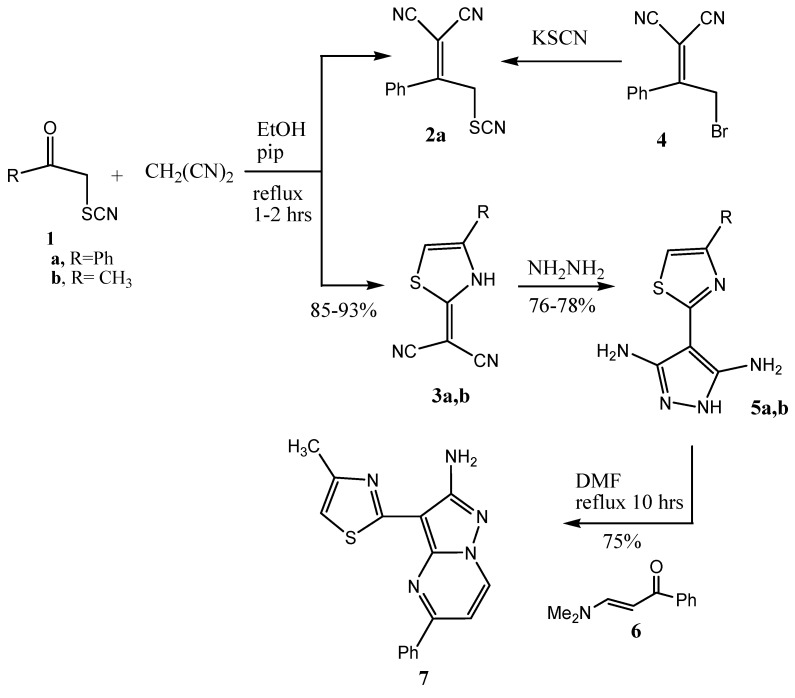
Syntheses of compounds **3a,b** and **7**.

Conflicting results have been reported for the structures of the products formed upon reaction of 2-thiocyanatoethanones (**1**) with active methylene nitriles. In 1986, Abdelrazek *et al* [1] proposed that reaction of **1a** with malononitrile in refluxing ethanol in the presence of catalytic piperidine affords the dicyanomethylidene adduct **2a**. Recently a patent described the formation of the thiazole **3a** from these substrates under very similar conditions (Scheme 1) [2]. Abdelrazek subsequently [3] reported that **2a** could also be produced by reaction of the brominated dicyanomethylidene **4a** with potassium thiocyanate, although a detailed procedure for this transformation was not provided. This observation served as evidence against the formation of **3a** as the product of the reaction of **1a** with malononitrile. Thiazole **3a** was also reported to form in the reaction between **1a** and malononitrile in presence of KOH [2,4].

## 2. Results and Discussion

**Figure 1:**
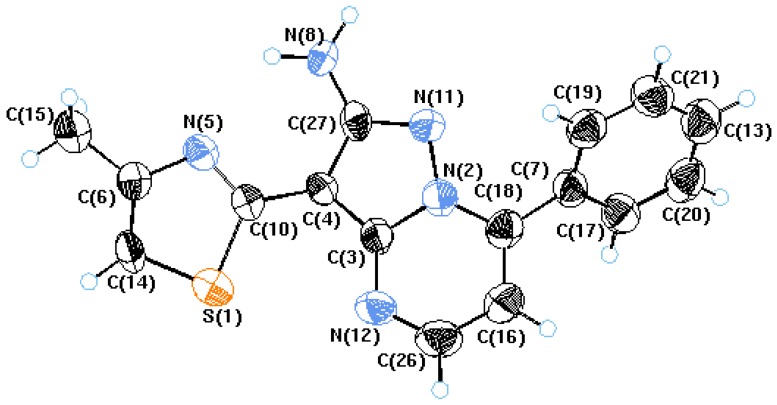
Plot of the X-ray crystallographic data of pyrazolo[1,a]pyrimidine **7**.

We recently [5] confirmed that in fact the product generated in the reaction of **1b** with malononitrile is **3b** by X-ray crystallographic analysis of the thiazolylpyrazolo[1,5-*a*]pyrimidine derivative **7** [6], which was produced *via* reaction of **3b** with hydrazine hydrate to yield the diaminopyrazole **5b** and subsequent reaction of **5b** with enaminone **6** (Figure 1).

Although the diaminopyrazole **5a**, produced by Salah Eldine [4] through the reaction of hydrazine hydrate with **3a**, proved to be identical with the substance we produced by reaction of **1a** with malononitrile, it was claimed that both **2** and **3** can be formed in this process. Consequently, we thought that a further effort aimed at confirming the structures of **5a**,**b** was in order since Abdelrazek has reported that **2a** can be utilized as a precursor in syntheses of several heterocyclic substances. Thus, if **2a** is really **3a** the structures of products claimed to be formed from **2a** in the earlier investigations need to be reassigned [1,3,7,8,9].

**Scheme 2:**
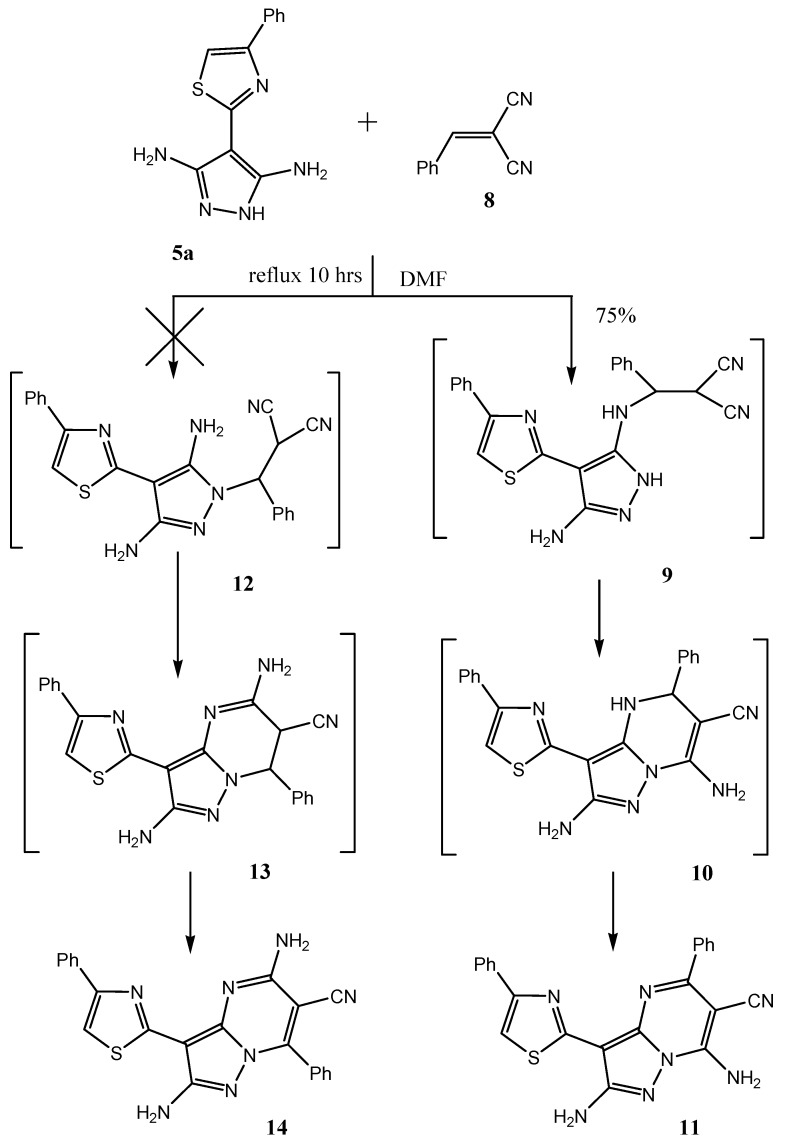
Syntheis of compound **11**.

In the current work, we explored the reaction of diaminopyrazole **5a** with benzylidenemalononitrile **8**. This process generates a product, resulting from sequential addition and molecular hydrogen elimination, that could be assigned as either **11** or **14**. These substances would be produced *via* the respective initially formed adducts **9 or 12** (Scheme 2).

Cyclization of these intermediates would form **10** or **13**, which then aromatize to generate **11** or **14**, respectively The actual structure of the final product was shown to be compound **11**, based on an analysis of ^15^N HMBC results. These results showed that the amino nitrogen at δ = 30 ppm is coupled to the bridgehead nitrogen at δ = 211 ppm.

**Scheme 3:**
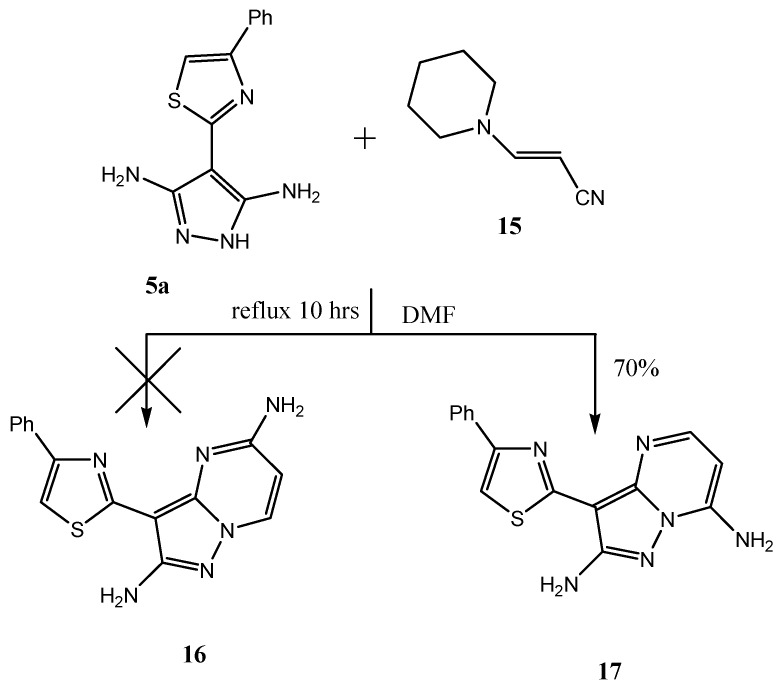
Syntheis of compound **17**.

**Figure 2:**
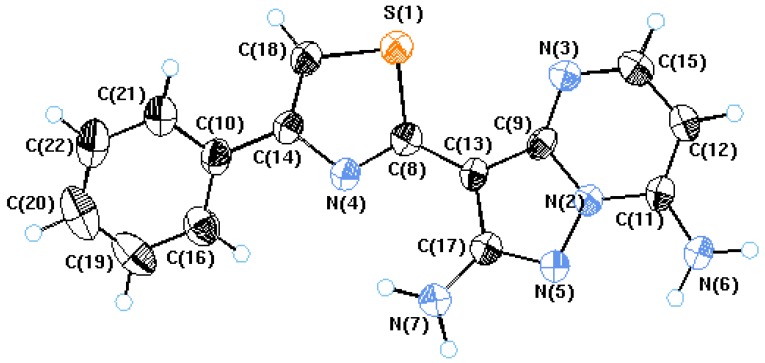
Plot of the x-ray crystallographic data of 3-(4-phenylthiazol-2-yl)pyrazolo[1,5-a]pyrimidine-2,7-diamine (**17**).

Diaminopyrazole **5a** reacts with enaminonitrile **15** to yield the pyrazolo[1,5-*a*] pyrimidinediamine **17** (Scheme 3). The isomeric adduct **16** was also excluded as the product based on the results of ^15^N HMBC and the X-ray crystallographic analyses (Figure 2) [10].

**Scheme 4:**
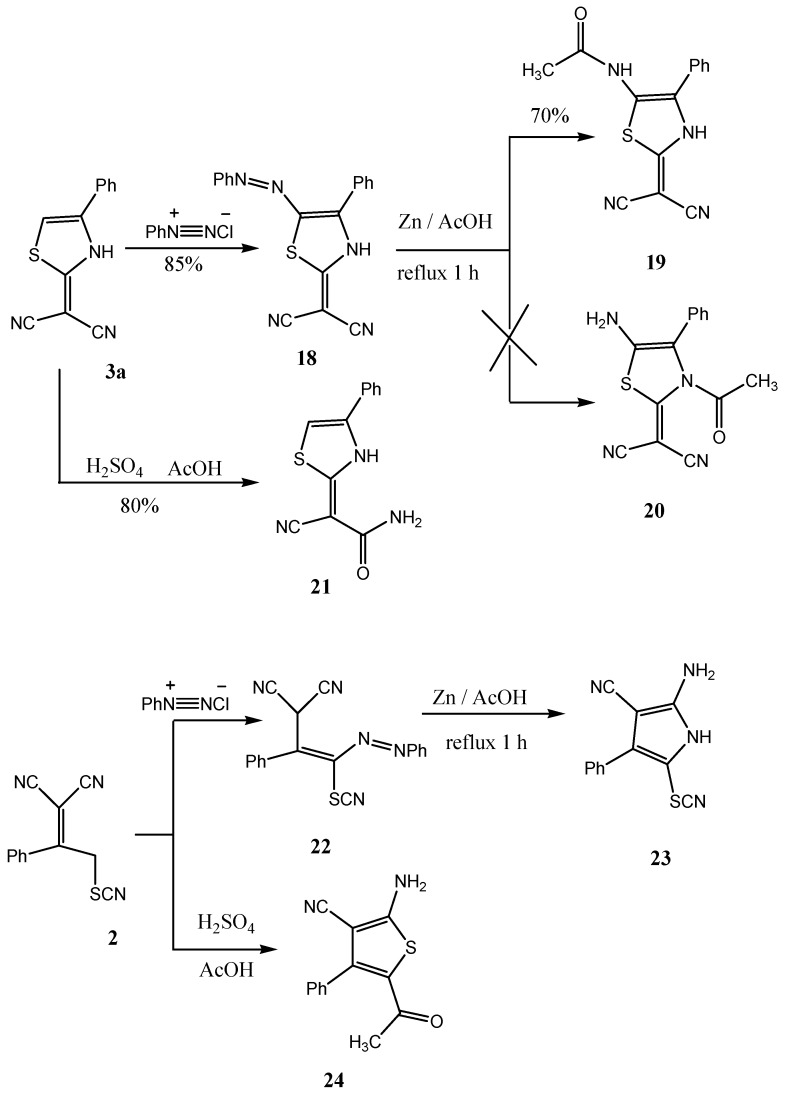
Syntheses of compound **19** and **21**.

The combined observations made in this investigation confirm the structural assignment of **3a**. Moreover, thiazole **3a** undergoes a coupling reaction with benzenediazonium chloride to yield the aryldiazo derivative **18**. Compound **18** was reduced by treatment with Zn metal in acetic acid to yield the amide **19** rather than the isomeric substance **20**. This assignment is based on the absence of amine signals in both the IR and ^1^H-NMR spectra of **19**. In addition, treatment of **3a** with sulfuric acid in acetic acid led to production of the amide **21**. Although **21** can exist in either a *E*- or *Z*-isomeric form, the *E*-stereoisomer appears to be generated selectively, as indicated from NOE difference experiments thus irradiating NH at δ = 12.46 ppm has enhanced amide NH_2_ at δ = 6.64 ppm and *vice versa*, such enhancement cannot occur with *Z*-isomeric. Clearly the claim [3] that pyrrole **23** and thiophene **24** are produced from a substance assumed to be **2** must be subjected to more concrete verification (Scheme 4).

**Scheme 5:**
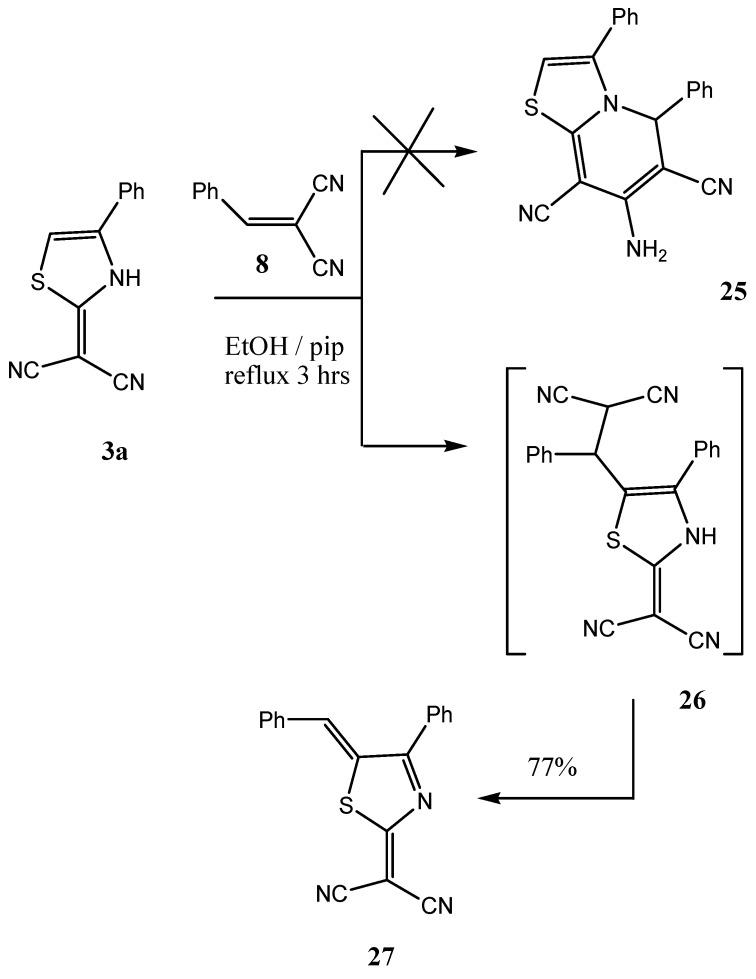
Synthesis of compound **27**.

Finally, in an attempt to extend the Salah Eldine [4] reaction of **3a** with furfurylidine malononitrile to its reaction with benzylidenemalononitrile **8**, only **27** was generated via the intermediacy of adduct **26**. The elimination of active methylene carbanions from substances analogous to **26** is well known [11] (Scheme 5).

**Scheme 6:**
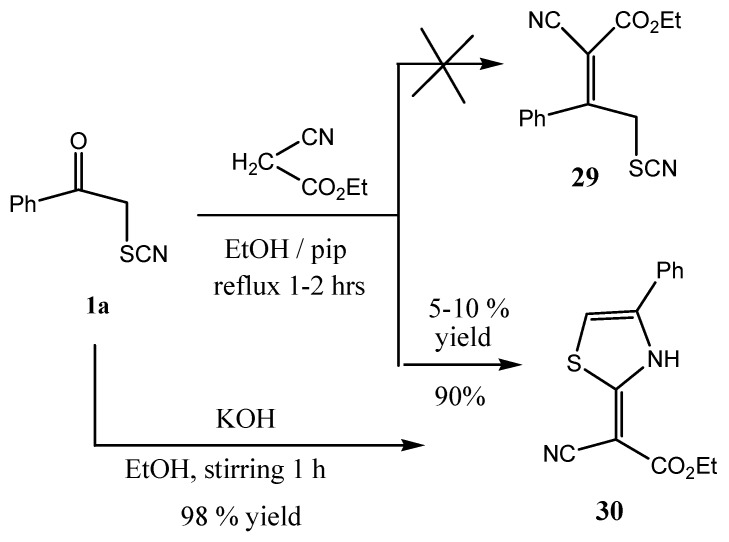
Synthesis of compound **30**.

Abdelrazek has also reported [12] that 1-phenyl-2-thiocyanatoethanone (**1a**) reacts with ethyl cyanoacetate to yield either the alkylidene or the thiazole adducts **29** or **30**, respectively. Again, analysis of the reaction mixture formed by carrying out this process under a variety of conditions revealed that **29** was not formed and only **30** was produced when potassium hydroxide was present (Scheme 6).

**Scheme 7:**
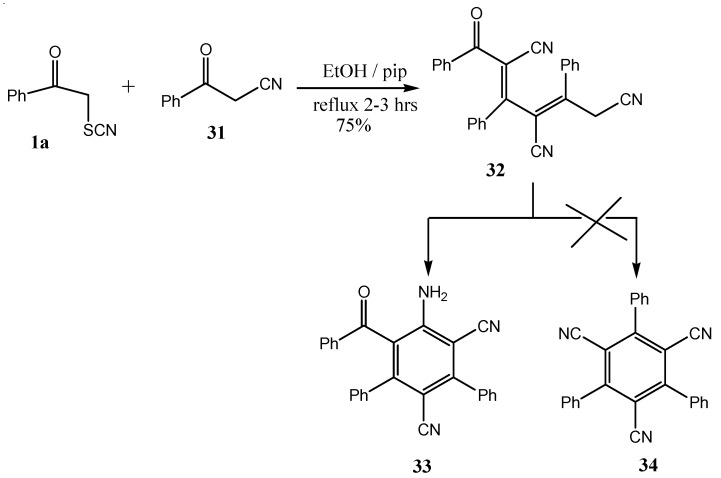
Synthesis of compound **33**.

Our attention next shifted to an exploration of condensation reactions of **1a** with other active methylene reagents. However, in our hands **1a** failed to produce adducts with acetylacetone, ethyl acetoacetate and the malononitrile dimer. under a variety of conditions reactions. Only the self tricondensation of **31** ocurred in the case of reaction of cyanoketone **31** with **1a**. Although the tricondensation product is well known, Briel *et al.* [13] recently assigned structure **32** to this substance. In contrast, Elnagdi *et al.* [14] have assigned the structure of this product as **33**, a substance which in fact can also be formed by cyclization of **32**. Again, Abdelrazek [15] has incorrectly claimed that this product is **33**. Of course, MS analysis shows that **33** differs from both **32** and **34** (Scheme 7). It is possible that Briel’s structural assignement is correct since heating the tricondensation product with zeolites affords an isomeric product that has been assigned as **33**.

**Scheme 8:**
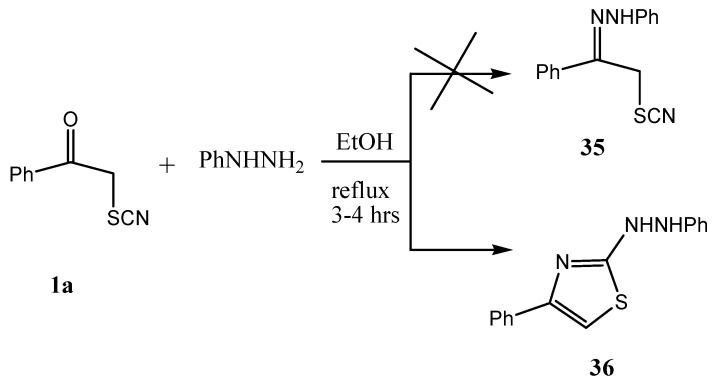
Synthesis of compound **36**.

We have also investigated the reactivity of **1a** with phenylhydrazine. This reaction has been reported by Abdelrazek *et al.* [8] to yield the hydrazone **35**. In our hands, only the thiazole **36** was isolated from the reaction mixture (Scheme 8).

## 3. Experimental

### 3.1. General

Melting points are reported uncorrected and were determined with a Sanyo (Gallaenkamp) instrument. Infrared spectra were recorded using KBr pellets and a Perkin-Elmer 2000 FT–IR instrument. ^1^H- and ^13^C-NMR spectra were determined by using a Bruker DPX instrument at 400 MHz for ^1^H-NMR and 100 MHz for ^13^C-NMR and either CDCl_3_ or DMSO*-d_6_* solutions with TMS as internal standards. Chemical shifts are reported in δ (ppm). Mass spectra were measured using VG Autospec Q MS 30 and MS 9 (AEI) spectrometer, with the EI (70 EV) mode. Elemental analyses were carried out by using a LEOCHNS-932 Elemental Analyzer.

### 3.2. General Procedure for the Syntheses of ***3a,b***

Solutions of malononitrile (0.66 g, 0.01 mol) and α-thiocyanatoketones **1a,b** (0.01 mol) in ethanol (15 mL) containing piperidine (5 drops) were stirred at reflux for 1–2 h. (completion assessed by TLC, 1:1 ethyl acetate-petroleum ether). The solid products, isolated by pouring the reaction mixtures into ice-water and subsequent separation by filtration, were crystallized from EtOH to afford green crystals.

*2-(4-Phenylthiazol-2(3H)-ylidene)malononitrile* (**3a**). Yield 93%; m.p. 275–276 °C; Anal. calcd. for C_12_H_7_N_3_S (225.27): C, 63.98; H, 3.13; N, 18.65; S, 14.23. Found: C, 63.94; H, 3.31; N; 18.45; S, 13.92; IR (KBr): υ_max_ 3147 (NH), 2210 (CN), 2175 (CN); ^1^H-NMR (DMSO): δ, ppm 7.33 (s, 1H, CH), 7.45–7.49 (m, 3H, Ar-H), 7.71–7.72 (m, 2H, Ar-H), 13.23 (br, 1H, NH, D_2_O exchangeable); ^13^C-NMR (DMSO): δ, ppm 172.27, 143.70, 130.01, 129.12(2C), 128.92, 127.51 (2C), 127.64, 117.75, 105.93 (2CN). MS: *m/z* (%) 225 (M^+^, 100), 180 (20), 134 (45), 108 (10), 102 (15), 89 (15), 77 (10).

*2-(4-Methylthiazol-2(3H)-ylidene)malononitrile* (**3b**). Yield 85%; m.p. 290–291 °C; Anal. calcd. for C_7_H_5_N_3_S (163.2): C, 51.52; H, 3.09; N, 25.75; S, 19.64. Found: C, 51.26; H, 3.12; N; 25.54; S, 19.27; IR (KBr): υ_max_ 3160 (NH), 2179 (2CN); ^1^H-NMR (DMSO): δ, ppm 2.17 (s, 3H, CH_3_), 6.71 (s, 1H, CH), 13.1 (br, 1H, NH, D_2_O exchangeable); ^13^C-NMR (DMSO): δ, ppm 170.10, 142.54, 105.93 (2CN), 95.23, 87.68, 16.54. MS: *m/z* (%) 163 (M^+^, 100), 136 (20), 118 (30), 98 (10), 71 (50).

### 3.3. General Procedure for the Syntheses of ***5a,b***

Mixtures of **3a,b** (0.01 mol) and hydrazine monohydrate (0.50 g, 0.01 mol) in DMF (10 mL) were stirred at reflux for 20 h. (completion assessed by TLC analysis using ethyl acetate-petroleum ether 1:1). The mixtures were cooled and poured into ice-water. The solid products, collected by filtration, were crystallized from DMF to give light yellow crystals.

*4-(4-Phenylthiazol-2-yl)-1H-pyrazole-3,5-diamine* (**5a**). Yield 78%; m.p. 322–323 °C; Anal. calcd. for C_12_H_11_N_5_S (257.31): C, 56.01; H, 4.31; N, 27.22; S, 12.41. Found: C, 55.80; H, 4.41; N; 26.88; S, 11.99; IR (KBr): υ_max_ 3372, 3256 (NH_2_), 3176, 3112 (NH_2_), 3132 (NH); ^1^H-NMR (DMSO): δ, ppm 5.39 (br, 4H, 2NH_2_, D_2_O exchangeable), 7.31–7.46 (m, 3H, Ar-H), 7.75 (s, 1H, CH), 7.96–7.98 (m, 2H, Ar-H), 10.73 (br, 1H, NH, D_2_O exchangeable); ^13^C-NMR (DMSO): δ, ppm 166.29, 162.08, 152.30 (2C), 134.31, 128.69 (2C), 127.69, 125.89 (2C), 107.48, 87.97. MS: *m/z* (%) 257 (M^+^, 100), 226 (10), 200 (5), 134 (35), 128 (10), 90 (10).

*4-(4-Methylthiazol-2-yl)-1H-pyrazole-3,5-diamine* (**5b**). Yield 76%; m.p. 330–332 °C; *Anal.* calcd. for C_7_H_9_N_5_S (195.24): C, 43.06; H, 4.65; N, 35.87; S, 16.42. Found: C, 42.89; H, 4.73; N; 35.60; S, 15.98; IR (KBr): υ_max_ 3371, 3275 (NH_2_), 3255, 3180 (NH_2_), 3118 (NH); ^1^H-NMR (DMSO): δ, ppm 2.23 (s, 3H, CH_3_), 5.62 (br, 4H, 2NH_2_, D_2_O exchangeable), 6.86 (s, 1H, CH), 10.67 (br, 1H, NH, D_2_O exchangeable); ^13^C-NMR (DMSO): δ, ppm 164.33, 161.56, 150.03 (2C), 106.88, 87.68, 16.84. MS: *m/z* (%) 195 (M^+^, 100), 164 (20), 138 (10), 123 (15), 112 (5), 72 (15).

### 3.4. Synthesis of 2,7-diamino-5-phenyl-3-(4-phenylthiazol-2-yl)pyrazolo[1,5-a]pyrimidine-6-carbonitrile *(**11**)*

A mixture of **5a** (2.57 g, 0.01 mol) and benzylidenemalononitrile (**8**, 1.54 g, 0.01 mol) in DMF (10 mL) was stirred at reflux for 10 h (completion assessed by TLC analysis using ethyl acetate-petroleum ether 1:1 as eluent). The reaction mixture was cooled and poured into ice-water giving a solid which was collected by filtration and crystallized from DMF to give a the product as yellow crystals in a yield of 75%; m.p. 298–300 °C; Anal. calcd. for C_22_H_15_N_7_S (409.47): C, 64.53; H, 3.69; N, 23.94; S, 7.83. Found: C, 64.61; H, 3.69; N; 23.79; S, 7.59; IR (KBr): υ_max_ 3430, 3318 (NH_2_), 3429, 3315 (NH_2_), 2211 (CN); ^1^H-NMR (DMSO): δ, ppm 6.85 (br, 2H, NH_2_, D_2_O exchangeable), 7.35–8.03 (m, 11H, Ar-H, thiazole-H), 8.57 (br, 2H, NH_2_, D_2_O exchangeable); ^13^C-NMR (DMSO): δ, ppm 195.47, 159.46, 157.98, 152.59, 148.91, 145.21, 137.10, 133.97, 130.37 (2C), 128.77 (2C), 128.70 (2C), 128.38 (2C), 127.89, 125.93, 116.48, 111.39, 92.24, 73.15. MS: *m/z* (%) 409 (M^+^, 100), 333 (5), 204 (15), 195 (5), 134 (30), 90(5).

### 3.5. Synthesis of 3-(4-phenylthiazol-2-yl)pyrazolo[1,5-a]pyrimidine-2,7-diamine *(**17**)*

A mixture of **5a** (2.57 g, 0.01 mol) and 3-(piperidin-1-yl)acrylonitrile (**15**, 1.36 g, 0.01 mol) in DMF (10 mL) was stirred at reflux for 10 h (completion assessed by TLC analysis using ethyl acetate-petroleum ether 1:1 as eluent). The reaction mixture was cooled and poured into ice-water giving a solid which was collected by filtration and crystallized from DMF to give a the product as yellow crystals in a yield of 70%; m.p. 329–330 °C; Anal. calcd. for C_15_H_12_N_6_S (308.36): C, 58.43; H, 3.92; N, 27.25; S, 10.40. Found: C, 58.33; H, 3.79; N; 27.20; S, 10.37; IR (KBr): υ_max_ 3443, 3305 (NH_2_), 3355, 3263 (NH_2_); ^1^H-NMR (DMSO): δ, ppm 6.13 (d, 1H, *J* = 6 Hz, CH), 6.62 (br, 2H, NH_2_, D_2_O exchangeable), 7.33–7.47 (m, 3H, Ar-Hs), 7.61 (br, 2H, NH_2_, D_2_O exchangeable), 7.85 (s, 1H, thiazole-H), 8.01 (m, 2H, Ar-Hs), 8.07 (d, 1H, *J* = 6 Hz, CH); ^13^C-NMR (DMSO): δ, ppm 160.59, 156.98, 152.22, 149.36, 147.14, 146.94, 134.31, 128.75 (2C), 127.72, 125.90 (2C), 109.66, 89.99, 88.59. MS: *m/z* (%) 308 (M^+^, 100), 268 (30), 175 (5), 154 (5), 134 (30), 102 (5), 89(5), 77 (5).

### 3.6. Synthesis of 2-(4-Phenyl-5-phenylazo-3H-thiazol-2-ylidene)malononitrile *(**18a**)*

A cold solution of benzenediazonium chloride (0.01 mol) was prepared by adding a solution of sodium nitrite (0.7 g in 10 mL H_2_O) to a cold solution of aniline hydrochloride (0.93 g, 0.01 mol of aniline in 5 mL concentrated HC1) with stirring at room temperature. The resulting solution was then added to cold solutions of **3a** (2.25 g, 0.01 mol) in ethanol (50 mL) containing sodium acetate (2 g). The reaction mixtures was stirred for 1 h and then filtered. The solid products were crystallized from EtOH to give the products as red crystals, yield 85%; m.p. 198–200 °C; Anal. calcd. for C_18_H_11_N_5_S (329.38): C, 65.64; H, 3.37; N, 21.26; S, 9.73. Found: C, 65.49; H, 3.51; N; 21.15; S, 9.39; IR (KBr): υ_max_ 3180 (NH), 2216 (2CN); ^1^H-NMR (DMSO): δ, ppm 5.03 (br, 1H, NH, D_2_O exchangeable), 7.25–7.60 (m, 8H, Ar-H), 8.22–8.24 (m, 2H, Ar-H); ^13^C-NMR (DMSO): δ, ppm 175.46, 147.29, 138.67, 131.79, 131.47, 130.73 (2C), 130.70 (2C), 129.59 (2C), 128.63 (2C), 127.05, 118.96, 117.71, 115.70 (2CN). MS: *m/z* (%) 329 (M^+^, 100), 301 (20), 237 (15), 225 (25), 153 (5), 103 (20), 92 (25), 77 (65).

### 3.7. Synthesis of N-(2-Dicyanomethylene-4-phenyl-2,3-dihydro-thiazol-5-yl)-acetamide *(**19**)*

A mixture of **18a** (3.29 g, 0.01 mol) and Zn dust (1 g) in AcOH (10 mL) was stirred at reflux for 1 hr (completion assessed by TLC analysis using ethyl acetate-petroleum ether 1:1 as eluent). The reaction mixture was cooled and poured into ice-water giving a solid which was collected by filtration and crystallized from EtOH to give the product as faint green coloured crystals in a yield of 70%; m.p. 335–338 °C; Anal. calcd. for C_14_H_10_N_4_OS (282.32): C, 59.56; H, 3.57; N, 19.85; S, 11.36. Found: C, 59.61; H, 3.44; N; 19.75; S, 11.42; IR (KBr): υ_max_ 3057 (NH), 3027 (NH), 2204 (CN), 2178 (CN); ^1^H-NMR (DMSO): δ, ppm 2.02 (s, 3H, CH_3_), 7.21–7.76 (m, 6H, Ar-Hs, NH, D_2_O exchangeable), 10.07 (br, 1H, NH, D_2_O exchangeable); ^13^C-NMR (DMSO): δ, ppm 169.86, 169.04, 164.93, 163.14, 128.69, 128.28 (2C), 128.09, 127.89 (2C), 127.73, 119.92, 117.67, 22.39. MS: *m/z* (%) 282 (M^+^, 60), 257 (20), 240 (100), 215 (85), 180 (10), 148(30), 121 (40), 104 (75), 93 (40), 77 (65), 73 (40).

### 3.8. Synthesis of 2-cyano-2-(4-phenylthiazol-2(3H)-ylidene)acetamide *(**21**)*

A mixture of **3a** (2.25 g, 0.01 mol) and H_2_SO_4_ (3 mL) in AcOH (10 mL) was stirred at reflux for 1 h. The reaction mixture was cooled and poured into ice-water giving a solid which was collected by filtration and crystallized from AcOH to give the product as white coloured crystals a yield of 80%; m.p. 229–231 °C; Anal. calcd. for C_12_H_9_N_3_OS (243.28): C, 59.24; H, 3.73; N, 17.27; S, 13.18. Found: C, 59.15; H, 3.58; N; 17.34; S, 13.32; IR (KBr): υ_max_ 3305, 3236 (NH_2_), 3126 (NH), 2184 (CN); ^1^H-NMR (DMSO): δ, ppm 6.64 (br, 2H, NH_2,_ D_2_O exchangeable), 7.15 (s, 1H, thiazole-H), 7.43–7.68 (m, 5H, Ar-H), 12.46 (br, 1H, NH, D_2_O exchangeable); ^13^C-NMR (DMSO): δ, ppm 169.77, 139.49, 129.37, 129.27, 128.93 (2C), 126.83 (2C), 126.07, 125.22, 118.94, 106.97. MS: *m/z* (%) 243 (M^+^, 90), 226 (100), 200 (45), 171 (5), 134 (40), 102 (45), 98 (10), 77 (5).

### 3.9. Synthesis of 2-(5-benzylidene-4-phenylthiazol-2(5H)-ylidene)malononitrile *(**27**)*

A mixture of **3a** (2.25 g, 0.01 mol) and benzylidenemalononitrile (**8**, 1.54 g, 0.01 mol) in EtOH (20 mL) in presence of piperidine (1 mL) was stirred at reflux for 3 h. The reaction mixture was cooled and poured into ice-water giving a solid which was collected by filtration and crystallized from EtOH to give the product as faint yellow coloured crystals in a yield of 77%; m.p. 280–282 °C; Anal. calcd. for C_19_H_11_N_3_S (313.38): C, 72.82; H, 3.54; N, 13.41; S, 10.23. Found: C, 12.94; H, 3.32; N; 13.31; S, 10.19; IR (KBr): υ_max_ 22.7 (CN), 2179 (CN); ^1^H-NMR (DMSO): δ, ppm 5.84 (s, 1H, CH), 7.15–7.42 (m, 10H, Ar-H); ^13^C-NMR (DMSO): δ, ppm 169.07, 141.21, 140.04, 131.24, 130.57, 129.69, 129.21 (2C), 128.91 (2C), 128.45 (2C), 128.21, 127.94, 127.61 (2C), 124.62, 122.00, 117.17. MS: *m/z* (%) 313 (M^+^, 80), 285 (10), 235 (90), 225 (100), 178 (10), 134 (60), 98 (20).

### 3.10. Synthesis of ethyl 2-cyano-2-(4-phenylthiazol-2(3H)-ylidene)acetate *(**30**)*

Solutions of ethyl cyanoacetate (1.13 g, 0.01 mol) and α-thiocyanatoketone **1a** (0.01 mol) in ethanol (15 mL) containing KOH (1 g) were stirred at room temperature for 1–2 h (completion assessed by TLC, 1:1 ethyl acetate- petroleum ether). The solid products, produced by pouring the reaction mixtures into ice-water containing HCl (2 mL) and subsequent separation by filtration, were crystallized from EtOH to give the product as white crystals in a yield of 90 %; m.p. 157–159 °C; Anal. calcd. for C_14_H_12_N_2_O_2_S (272.32): C, 61.75; H, 4.44; N, 10.29; S, 11.77. Found: C, 61.71; H, 4.32; N; 10.18; S, 11.98; IR (KBr): υ_max_ 3152 (NH), 2211 (CN); ^1^H-NMR (DMSO): δ, ppm 1.23 (t, 3H, *J* = 6 Hz, CH_3_), 4.16 (q, 2H, *J* = 6 Hz, CH_2_), 7.15 (s, 1H, thiazole-H), 7.43–7.71 (m, 5H, Ar-H), 12.99 (br, 1H, NH, D_2_O exchangeable); ^13^C-NMR (DMSO): δ, ppm 169.23, 166.55, 140.68, 129.31 (2C), 128.75, 128.75 (2C), 127.00, 117.54, 107.49, 62.93, 59.68, 14.55. MS: *m/z* (%) 272 (M^+^, 90), 226 (100), 200 (50), 172 (10), 153 (5), 134 (50), 102 (40), 89 (10), 77 (10).

### 3.11. Synthesis of 1-phenyl-2-(4-phenylthiazol-2-yl)hydrazine *(**36**)*

A mixture of **1a** (1.77 g, 0.01 mol), phenyl hydrazine (1.80 g, 0.02 mol), in EtOH (20 mL) was refluxed for 3–4 h (completion assessed by TLC analysis using ethyl acetate-petroleum ether 1:1 as eluent). The reaction mixture was cooled and poured into ice-water giving a solid which was collected by filtration and crystallized from EtOH to give the product as white crystals in a yield of 75%; m.p. 216–218 °C; *Anal.* calcd. for C_15_H_13_N_3_S (267.35): C, 67.39; H, 4.90; N, 15.72; S, 11.99. Found: C, 67.17; H, 4.88; N; 15.58; S, 12.12; IR (KBr): υ_max_ 3239 (NH), 3089 (NH); ^1^H-NMR (DMSO): δ, ppm 6.47–7.61 (m, 11H, Ar-H, thiazole-H), 9.08 (br, 1H, NH, D_2_O exchangeable), 12.60 (br, 1H, NH, D_2_O exchangeable); ^13^C-NMR (DMSO): δ, ppm 163.35, 147.00, 130.91, 128.94 (2C), 128.54 (2C), 128.01, 127.91, 126.59 (2C), 119.59, 112.59 (2C), 111.19. MS: *m/z* (%) 267 (M^+^, 100), 234 (5), 207 (5), 175 (15), 148 (5), 117 (70), 93 (50), 77 (10).

## 4. Conclusions

In conclusion, the results of this study confirm that the substance thought to be 2-(1-phenyl-2-thiocyanatoethylidene)malononitrile (**2**) is not generated in the reaction of ketone **1a** with malononitrile. The product produced in this process is in fact the 2-(thiazol-2(3*H*)-ylidene)-malononitriles **3a,b**. If 2-(1-phenyl-2-thiocyanatoethylidene)-malononitrile (**2**) was really produced in this reaction, then an exact experimental procedure that would enable a reasonably skilled chemist to repeat the process should be published. Finally, this effort has provided the structures of some products arising from reactions of **3a,b** with a variety of electrophilic and nucleophilic reagents.
